# Quality of childcare and delayed child development in left-behind children in China

**DOI:** 10.1038/s41390-023-02840-7

**Published:** 2023-10-16

**Authors:** Kelly Lin, Yu-ming Zhou, Hai-ping Ma, Fan He, Xiao-na Huang, Xiao-bo Tian, Yi Zheng, Jing Sun

**Affiliations:** 1 Institute of Integrated Intelligence and Systems, and School of Medicine and Dentistry, Griffith University, Gold Coast, QLD Australia; 2 Beijing Anding Hospital, Capital Medical University & The National Clinical Research Center for Mental Disorders & Beijing Key Laboratory of Mental Disorders, Beijing Institute for Brain Disorders, Beijing, People’s Republic of China; 3 Youfu Hospital of Binzhou City, No. 585, Changjiang 1st Road, Bincheng District, Binzhou City, Shandong Province People’s Republic of China; 4 United Nations Children’s Fund (UNICEF China Office), Beijing, People’s Republic of China

## Abstract

**Background:**

Inequalities in job opportunities and income prompts many Chinese parents to leave rural regions to work in urban regions. Their children are left behind in rural regions, subjected to worse quality of childcare that jeopardizes their development. This study aimed to examine the association between quality of childcare and delayed child development in under-three years children left behind in China.

**Methods:**

Cross-sectional national survey was conducted in children left behind in rural China in 2017. Exploratory and confirmatory factor analysis was used to develop a quality of childcare index. Mutlilevel analyses determined factors associated with quality of childcare and child development on a province and individual level.

**Result:**

The largest population of at-risk children left behind were found in higher-GDP provinces. Children left behind had the lowest mean quality of childcare score. Multilevel analysis found that province level accounted for a great proportion of variance observed.

**Conclusions:**

While migration to urban regions for work may improve household income, a trade-off in worse quality of childcare and developmental delays exists. With improving household income often being the greatest contributing factor for parental migration, policies to reduce inequalities in job opportunities and wealth between rural and urban regions are required.

**Impact:**

- Previous studies identified higher prevalence of developmental delays in children left behind in China. However, quality of childcare has not been examined.
- Based on WHO’s Nurturing Care Framework, we developed a quality of childcare index to assess its association with child development in children left behind.
- Greatest proportion of children left behind at-risk of developmental delays resided in higher-GDP states, indicating a trade-off in worse quality of childcare and developmental delays.
- Since improving household income is the main factor for parental migration, policies to close inequalities in job opportunities and wealth between rural and urban regions are required.

## Introduction

China remains a country with significant wealth inequality, especially between urban and rural regions.^1^ Rapid developments in Chinese cities have produced many job opportunities with better pay, attracting migrant workers from rural regions.^2^ However, restrictions in migration from the household registration system in China means that children are often left behind in rural regions. Thus, despite economic gains, children left behind by migrant workers face significant disadvantages and risk factors that hinder their development and wellbeing.^3^ In 2015, UNICEF estimated that 68.77 million of children were left behind in rural Chinese regions by parents who migrate for work.^4^ The population of children left behind accounted for 29.4% of all rural children, and in some provinces, accounting for up to 40% of the rural children population, highlighting the magnitude of the issue.^4^

Almost 20% of children left behind were separated from their parents before 1 year of age and are left to adapt to changes in internal family structure and emotional difficulties that result from being separated with parents.^5^ Lack of parental involvement in addition to residing in rural regions with the greatest level of poverty make children left behind one of the most disadvantaged and marginalized population at risk for developmental delays.

The importance of healthy development is further emphasized by the World Health Organization (WHO) as a crucial component in achieving Sustainable Development Goals as all children should be able to reach their full potential to become healthy productive adults.^6,7^ The Nurturing Care Framework has subsequently been established by the WHO, outlining five components for healthy early childhood development, including adequate nutrition, opportunities for early learning, security and safety, responsive caregiving, and good health.^7^

In children left behind with absent parents, it is difficult to receive the emotional and physical interactions required to achieve the nurturing care required for health development. Children who are left behind with long-term parental–child separation often have minimal communication with their parents about their emotional state reflecting poor responsive caregiving and thus often precipitate psychosocial problems.^8^

Physical stimulation are linked to early learning opportunities where a stimulating environment with age-appropriate toys, books, and positive interactions such as praising children help children achieve health development.^9^ However, early learning opportunities through simple activities including playing, reading, counting, and singing with children can also be limited for children left behind.^6,10^ Adequate nutrition is also required to ensure healthy brain development in the first three years of life.^11^ Nutrition deficiencies limits brain metabolism and thus development, which may result in long-term dysfunctions that manifests in developmental disabilities.^11^ Previous studies have identified higher rates of malnutrition in children left behind in the form of stunting and underweight, reflecting poor nutrition. Another critical aspect of nurturing care that threatens the wellbeing of children left behind is poor safety measures around the household and surrounding environment, which results in higher rates of unintentional injury such as burns, mechanical injury and poisons.^12^

A large proportion of children that are left behind by both parents have older grandparents as their primary caregivers.^13^ Older grandparents often lack the knowledge about the importance of stimulating play and positive parenting practices, leaving the children to play by themselves or watch television alone.^14^ Similarly, older grandparents that are often the main caregivers have limited physical strength and safety knowledge on emergency services and environmental hazard, hindering them from providing effective supervision, contributing to higher rates of unintentional injury.^15^

With absent parents, limitations in each aspect of the Nurturing Care Framework prevent children left behind from receiving adequate care required for healthy development. Thus, it is expected that children who are left behind would be at high risk of developmental delays. Although previous studies have identified developmental delays in children left behind, quality of childcare has been less examined. Given the importance of nurturing care, this study aimed to fill this research gap by developing a quality of childcare index based on WHO’s Nurturing Care Framework.

## Methods

### Study design

This cross-sectional study used random sampling to collect data from November 2016 to January 2017 in 11 provinces and 1 municipality with low gross domestic product (GDP) in rural areas across China, among which 24 counties and 40 villages were randomly selected. Multilevel clustered random sampling method was used to select sample. The 12 provinces/municipality were selected because they had a high population of children left behind and provided an unique research opportunity to investigate the development of children left behind. A total of 4976 children were recruited, with 2515 children left behind and 2461 children raised by both parents. In this study, children left behind were identified as those who are left behind with one parent or those that are left behind by both parents. All children included were divided into left behind or non-left behind. This dataset included only children 3 years of age and under.

All healthcare professionals participating in data collection were trained by the United Nation’s Child Fund assigned project experts prior to data collection. Trained local health workers identified children who met the inclusion criteria and briefed each household on the questionnaire. Family information questionnaire including questions that compromised the Quality of Childcare Index were then answered by caregivers independently at home. Thus, data collectors were unaware of the status of the children left behind or not. As a result of the quality of training, there is <2% missing data.

### Measures

#### Quality of childcare

Assessment of quality of childcare were made based on four domains in line with WHO’s Framework of Nurturing Care.^7^ This includes psychosocial stimulation in relation to early education, exposure to environmental hazard, knowledge of emergency services systems and nutrition. Dataset was coded in which higher scores indicated better quality of childcare. Healthcare was not assessed as initial analysis on child immunization showed that most children left behind (91.9%) and not left behind (91.9%) are up to date with the national pediatric immunization schedule with no significant difference (*p* = 0.977), indicating equal and adequate access to healthcare.

Psychosocial stimulation was assessed based on seven items with answers “yes” or “no”: (1) homemade toys available, (2) store bought toys available, (3) child plays with household items such as pots, (4) primary caregiver engages in reading, counting, drawing with child, (5) primary caregiver reads stories or sings to child, (6) primary caregiver take child out for walks, (7) primary caregiver play games such as hide and seek with child. Exposure to environmental hazards was assessed with seven items with responses “yes” or “no”: (1) primary caregiver accompanies child to nearby rivers and lakes for recreational water activities, (2) child goes to nearby rivers and lakes for recreational water activities with other non-adults (under 18), (3) child bath alone in bath tub, (4) child play in kitchen and/or bathroom, (5) matches and lighters are placed within child’s reach, (6) hot water or food are placed within child’s reach, (7) storage of petrol and other flammables indoors. Access and exposure to each environmental hazard is scored as 0, to match the directionality of other factors assessed. Thus, higher score in the environmental hazard domain indicates less exposure to environmental hazard and better quality of childcare. Knowledge of emergency services are assessed based on whether primary caregivers know what number to dial for (1) police, (2) fire, (3) ambulance in an emergency. Outcomes were measured with three responses and scored from 0 to 2, respectively, cannot provide a number, provides an incorrect number, and provides the correct number.

Child nutrition is scored based on exposure to six food groups, with answers “yes” or “no”: (1) dairy, (2) carbohydrates, (3) vitamin A-rich foods, (4) eggs, (5) poultry or fish, (6) beans and legumes. To ensure equal assessment of nutritional diversity among children of all ages, only children 6 months (M) and older were included when determining quality of childcare, as the recommended age to begin solid foods for children are 4–6 M.^16,17^ Age-appropriate food choices are included in each food group. For assessment of dairy consumption, this includes formula, cow’s milk, and all other liquid dairy products such as drinking yoghurt and goat’s milk. For assessment of carbohydrates, foods such as infant rice cereal and rice puree were included to account for younger children starting solid food.

#### Child development

Child development was assessed using a brief Ages and Stages Questionnaires (ASQ-3) in a rural setting as an alternative to the comprehensive ASQ used in clinical settings to screen for children at risk of developmental delays. Four questions has been used as a brief assessment rather than the original 25 questions for ease of administration in rural settings. The reliability and validity of the questionnaire has been assessed with a sensitivity of 75% and specificity of 86% in detecting developmental delays.^18,19^ The four questions used in the brief ASQ has been identified and verified through a pilot study on under 3 children in China, achieving a high predictive value and reliability of Cronbach’s $\alpha$ of 0.90. Development of each child was assessed using four questions in relation to their age. To compare the association of quality of childcare on child development at different stages, we have divided the sample into three groups—under 12 M of age, between 12 M to 23 M of age, and 24 M to 36 M of age. Total 36 M and under development were also assessed. To further compare differences between developmental domains, included questions were divided into domains of motor, communication, and overall development. This was also assessed across four age groups. Children that are unable to complete any one of the four age-specific tasks specified by the questionnaire are classified as “high risk”, while children that are able to complete all four tasks are classified as “normal development”. The differences between “high-risk” left behind children (LBC) and non-LBC will be assessed.

#### Other variables

Association between demographic variables, child development, and quality of childcare were analyzed to further identify risk factors. Household income was divided in four brackets (<2000 yuan, 2000–5999 yuan, 6000–10,000 yuan, and >10,000 yuan). Main source of household income was also assessed (farming, business, casual/part time jobs, and others). Highest education of primary caregiver included three categories (no education, primary, and secondary and above).

#### Wealth level of provinces and municipality

Eleven provinces and one municipality included were divided into higher-GDP, middle-GDP, and lower-GDP based on their GDP to compare the differences in proportion of LBC, quality of childcare, and the presence of developmental delays.^20^ Four provinces or municipality with the highest GDP were classified as higher-GDP (Chongqing, Hubei, Shaanxi, Hunan), followed by the next four provinces or municipality as middle-GDP (Hebei, Henan, Jiangxi, Sichuan). The four provinces (Anhui, Guangxi, Shanxi, Guizhou) or municipality with the lowest GDP are then classified as lower-GDP.

### Statistical analysis

Chi-square analysis was first conducted to identify differences in demographic factors between children left behind and not left behind. Differences in demographic factors and presence of children left behind across all 11 provinces and municipals included were also assessed using Chi-square analysis. To devise a quality of childcare index, exploratory and confirmatory factor analysis (CFA) was conducted. Adequate model fit was judged by indices of fitting including Comparative Fit Index (CFI) $\ge$ 0.90, Goodness-of-Fit index (GFI) $\ge$ 0.95 and Root Mean Square Error of Approximation (RMSEA) $<$ 0.08. Reliability of the index was further assessed based on Cronbach’s $\alpha$ levels, with overall >0.70 indicating good reliability. The final scale composed of 20 items was formed. Based on the Exploratory Factor Analysis (EFA), factors with poor fit and an eigenvalue <1 was excluded. Thus, a 20-item scale was formed to assess quality of childcare. Two items related to water recreational activities was excluded from the exposure to environmental hazards subscale, while dairy was excluded from the nutritional subscale.

Multilevel analysis with a two-level hierarchy (individual child and province) was then conducted based on the index created to determine the association between quality of childcare and child development. Association between primary caregiver, age of primary caregiver, highest education of primary caregiver, main source of household income, level of household income, status as children left behind and quality of childcare was also assessed using multilevel analysis.

## Results

### Differences between LBC and non-LBC

Table 1 presents the demographic differences between children left behind and those who are currently raised by both parents. Significant differences existed across all factors assessed. Most children raised by both parents (98.3%) had their parents as their primary caregiver as compared to children left behind (63.9%), while around 35.5% of children left behind had their grandparents as their primary caregiver. The main source of household income also differed. Most families of children left behind (91.2%) relied on casual and part time jobs as their main source of income, while the sources of income were more distributed for children raised by both parents.

**Table 1:** Left behind child demographic factors.

		Not left behind	Left behind	% Difference	
Primary caregiver	Parents (%)	2419 (98.3)	1607 (63.9)	34.4	<0.001
	Grandparents (%)	42 (1.7)	894 (35.5)	−33.8	
	Other adult relatives (%)	0 (0)	13 (0.5)	−0.5	
	Other under 18 relatives (%)	0 (0)	13 (0)	0	
				0	
Child age range	3 M (%)	209 (13.5)	158 (8.9)	4.6	0.002
	6 M (%)	111 (7.2)	111 (6.3)	0.9	
	8 M (%)	170 (11.0)	192 (10.9)	0.1	
	12 M (%)	258 (16.7)	295 (16.7)	0	
	18 M (%)	245 (15.8)	299 (16.9)	−1.1	
	24 M (%)	253 (16.3)	345 (19.5)	−3.2	
	30 M (%)	267 (17.2)	327 (18.5)	−1.3	
	36 M (%)	35 (2.3)	42 (2.4)	−0.1	
Primary caregiver highest education	No education (%)	20 (5.3)	743 (20.8)	−15.5	<0.001
	Primary (%)	83 (21.9)	1589 (44.5)	−22.6	
	Secondary or above (%)	276 (72.8)	276 (34.7)	38.1	
Main source of household income	Farming (%)	539 (21.9)	109 (4.3)	17.6	<0.001
	Business (%)	428 (17.4)	84 (3.3)	14.1	
	Casual/part time jobs (%)	1042 (42.4)	2293 (91.2)	−48.8	
	Other (%)	451 (18.3)	29 (1.2)	17.1	
Household income	<2000 Yuan (%)	191 (7.8)	167 (6.7)	1.1	<0.001
	2000–5999 Yuan (%)	1154 (47.1)	1176 (46.9)	0.2	
	6000–10,000 Yuan (%)	535 (21.9)	730 (29.1)	−7.2	
	>10,000 yuan (%)	568 (23.2)	436 (17.4)	5.8	
Risk for developmental issues	Normal development (%)	(89.6)	(89.0)	0.6	0.611
	High risk (%)	160 (10.4)	192 (11.0)	−0.6	
Mean total quality of childcare score		17.0 (3.7)	16.0 (4.0)	1	0.002
Mean psychosocial stimulation score		4.1 (1.8)	3.9 (1.8)	0.2	0.003
Mean environmental hazard score		4.6 (0.8)	4.5 (0.9)	0.1	0.003
Mean emergency service knowledge score		5.2 (1.8)	4.5 (2.3)	0.7	<0.001
Mean nutrition score		3.3 (1.6)	3.2 (1.4)	0.1	0.092

Average quality of childcare score were significantly lower across all aspects for children left behind as shown in Table 1. The average 20-item quality of childcare index was created through exploratory and confirmatory factor analysis, with adequate reliability as displayed in Table 2. Children who were left behind were subjected to lower psychosocial stimulation (Mean 3.9, SD 1.8), more environmental hazards (Mean 4.5, SD 0.9) and worse overall childcare (Mean 16.0, SD 4.0). Primary caregivers of children left behind (Mean 4.5, SD 2.3) also had worse knowledge in which emergency service number to dial. Table 3 provides further information on quality of childcare received by children left behind with one parent or grandparents and relatives (no parents). Other than nutrition, children raised by both parents (Total Mean 17.0, SD 3.7) received the highest quality of care followed by children left behind with one parent (Total Mean 16.4, SD 3.8). Children left behind with grandparents or other relatives, without parents at home scored the lowest across all components of childcare assessed (Total Mean 14.9, SD 4.3).

**Table 2:** Differences between demographic factors between each province.

		High					Middle					Low					
		Chongqing	Hubei	Shaanxi	Hunan	Total	Hebei	Henan	Jiangxi	Sichuan	Total	Anhui	Guangxi	Shanxi	Guizhou	Total	
Left behind children	Not left behind (%)	261 (39.7)	131 (39.0)	217 (64.2)	168 (32.2)	777 (41.9)	268 (86.7)	152 (61.0)	317 (47.7)	120 (46.3)	857 (57.8)	296 (38.2)	34 (52.3)	79 (43.6)	417 (67.3)	826 (50.4)	<0.001
	Left behind (%)	396 (60.3)	205 (61.0)	121 (35.8)	354 (67.8)	1076 (58.1)	41 (13.3)	97 (39.0)	348 (52.3)	139 (53.7)	625 (42.2)	478 (61.8)	31 (47.7)	102 (56.4)	203 (32.7)	814 (49.6)	
High-risk left behind children (%)		7 (3.4)	20 (16.3)	24 (33.8)	9 (13.4)	60 (14.5)	0 (0.0)	14 (15.2)	36 (15.2)	7 (8.2)	57 (14.5)	13 (4.2)	1 (100)	17 (28.3)	6 (2.9)	37 (9.3)	<0.001
High-risk non-left behind children (%)		0 (0.0)	3 (6.3)	20 (13.2)	6 (6.5)	29 (7.4)	0 (0.0)	32 (20.9)	41 (14.2)	14 (12.6)	87 (13.5)	23 (8.6)	1 (100)	5 (10.0)	16 (43.2)	45 (10.7)	<0.001
Primary caregiver	Parents (%)	527 (80.1)	289 (86.0)	527 (94.7)	276 (52.8)	1619 (74.4)	305 (98.4)	236 (94.4)	549 (82.6)	187 (72.2)	1277 (86.05)	623 (80.6)	53 (81.5)	155 (85.6)	506 (81.7)	1337 (81.6)	<0.001
	Grandparents (%)	130 (19.8)	47 (14.0)	130 (5.3)	243 (46.5)	550 (25.3)	5 (1.6)	14 (5.6)	115 (17.3)	70 (27.0)	204 (13.75)	146 (18.9)	12 (18.5)	26 (14.4)	111 (17.9)	295 (18.0)	
	Other adult relatives (%)	2 (0.2)	0 (0.0)	1 (0.0)	3 (0.6)	6 (0.3)	0 (0.0)	0 (0.0)	1 (0.2)	2 (0.8)	3 (0.02)	4 (0.5)	0 (0.0)	0 (0.0)	2 (0.3)	6 (0.4)	
	Other under 18 relatives (%)	0 (0.0)	0 (0.0)	0 (0.0)	1 (0.2)	1 (0.0)	0 (0.0)	0 (0.0)	0 (0.0)	0 (0.0)	0 (0.0)	0 (0.0)	0 (0.0)	0 (0.0)	0 (0.0)	0 (0.0)	
Primary caregiver highest education	No education (%)	55 (12.9)	77 (24.8)		30 (6.1)	162 (14.6)	20 (9.3)	28 (9.3)	87 (18.5)	163 (42.7)	298 (23.08)	124 (36.6)	33 (16.3)	5 (2.6)	99 (27.7)	261 (23.9)	<0.001
	Primary (%)	170 (47.6)	99 (31.9)		212 (43.3)	481 (43.2)	110 (51.2)	110 (51.2)	231 (49.0)	129 (33.8)	580 (44.93)	120 (35.4)	105 (52.0)	96 (49.0)	170 (47.6)	491 (44.9)	
	Secondary or above (%)	88 (24.6)	134 (43.2)		248 (50.6)	470 (42.2)	85 (39.5)	85 (39.5)	153 (32.5)	90 (23.6)	413 (31.99)	95 (28.0)	64 (31.7)	95 (48.5)	88 (24.6)	342 (31.3)	
Main source of household income	Farming (%)	19 (2.9)	13 (3.9)	24 (7.1)	56 (10.7)	112 (6.1)	31 (10.0)	120 (48.0)	27 (4.1)	93 (36.0)	271 (18.3)	107 (13.8)	22 (33.8)	38 (21.0)	99 (16.0)	266 (16.2)	<0.001
	Business (%)	44 (6.7)	24 (7.1)	53 (15.7)	49 (9.4)	170 (9.2)	15 (4.8)	9 (3.6)	113 (17.0)	11 (4.1)	148 (10.0)	135 (17.4)	5 (7.7)	4 (2.2)	50 (8.1)	194 (11.8)	
	Casual/part time jobs (%)	530 (80.7)	290 (86.4)	203 (60.1)	378 (72.4)	1401 (75.6)	252 (81.3)	115 (46.0)	432 (65.1)	145 (56.2)	944 (63.7)	509 (65.8)	35 (53.8)	131 (72.4)	314 (50.6)	989 (60.3)	
	Other (%)	64 (9.7)	9 (2.7)	58 (17.2)	39 (7.5)	170 (9.2)	12 (3.9)	6 (2.4)	92 (13.9)	9 (3.5)	119 (8.0)	23 (3.0)	3 (4.6)	8 (4.4)	157 (23.5)	191 (11.7)	
Household income	<2000 Yuan (%)	14 (2.1)	7 (2.1)	47 (14.0)	55 (0.5)	123 (6.7)	25 (8.1)	23 (9.2)	26 (4.0)	53 (20.6)	127 (5.0)	37 (4.8)	9 (13.8)	46 (25.6)	17 (2.8)	109 (6.7)	<0.001
	2000–5999 Yuan (%)	267 (40.8)	89 (26.6)	141 (42.0)	236 (45.0)	733 (39.7)	1311 (81.0)	146 (58.2)	350 (53.3)	156 (60.7)	1963 (78.0)	296 (38.3)	31 (47.7)	122 (67.8)	245 (39.7)	694 (42.4)	
	6000–10,000 Yuan (%)	202 (30.9)	138 (41.3)	79 (23.5)	111 (21.2)	530 (28.7)	34 (11.0)	55 (21.9)	131 (19.9)	19 (7.4)	221 (8.8)	276 (35.7)	13 (20.0)	9 (5.0)	199 (32.3)	497 (30.4)	
	>10,000 yuan (%)	171 (26.1)	100 (29.9)	69 (20.5)	122 (23.3)	462 (25.0)	0 (0.0)	27 (10.8)	150 (22.8)	29 (11.3)	206 (8.2)	164 (21.2)	12 (18.5)	3 (1.7)	156 (25.3)	335 (20.5)

**Table 3:** Mean quality of childcare score of left behind and non-left behind children.

	Not left behind (A)	Left with one parent at home (B)	Left with no parents at home (C)	*p* value	Post hoc
Mean total quality of childcare score	17.0 (3.7)	16.4 (3.8)	14.9 (4.3)	<0.001	A > B*** A > C*** B > C***
Mean psychosocial stimulation score	4.07 (1.8)	3.9 (1.8)	3.8 (1.7)	0.005	A > B* A > C*
Mean environmental hazard score	4.6 (0.8)	4.5 (0.9)	4.4 (1.0)	0.003	A > C*** B > C*
Mean emergency service knowledge score	5.2 (1.8)	4.8 (2.1)	3.5 (2.6)	<0.001	A > B*** A > C*** B > C***
Mean nutrition score	3.3 (1.6)	3.1 (1.5)	3.3 (1.4)	0.153	

*p < 0.05,

***p < 0.001

### Differences between provinces

Twelve provinces/municipality were sampled in this study and divided into three levels based on their GDP.^20^ The lower GDP provinces include Anhui, Guangxi, Shanxi, and Guizhou. The middle-GDP provinces include Hebei, Henan, Jiangxi, and Sichuan; while the higher-GDP provinces include Chongqing, Hubei, Shaanxi, and Hunan. The highest percentage of children left behind was found in Hunan (67.8%) followed by Anhui (61.8%) and Hubei (61.0%). Based on GDP classification, higher-GDP provinces (58.1%) had the greatest proportion of children left behind at-risk of developmental delays (15.1%) as compared to children left behind in lower-GDP province (9.3%). In terms of main source of household income, most households from higher-GDP provinces depended on casual or part-time jobs (75.6%) as compared to middle-income (63.7%) and lower-GDP (60.3%) provinces.

### Exploratory and confirmatory analysis

Exploratory factor analysis (EFA) and confirmatory factor analysis (CFA) were conducted to create a quality of childcare index. Maximum likelihood analysis with direct oblimin rotation for Chinese children and their caregiver based on their answers to the administered survey are presented in Table 3. Exploratory factor analysis found a total of four factors with an eigenvalue greater than one. Based on the results, items 1 to 7 were loaded as psychosocial stimulation factor, items 8 to 14 are loaded as exposure to environmental hazards, items 15 to 17 are loaded as knowledge of emergency services and items 18 to 23 were loaded as nutrition factor. Model fit and factor structure for the identified four factor model were then examined using CFA using Amos. Goodness of fit assessed based on model fit was adequate with a comparative fit index (CFI) of 0.903, Tucker Lewis index TLI of 0.890 and root mean square error of approximation (RMSEA) of 0.046. The reliability of the index created was also reasonable with a total Cronbach’s *α* of 0.725, 0.674 for psychosocial stimulation, 0.532 for exposure to environmental danger, 0.875 for knowledge of emergency services, and 0.669 for nutrition subindex.

### Multilevel analysis

Results from multilevel analysis presented in Table 4 indicated that although total quality of childcare score was not associated child development, aspects of childcare was significantly associated with different domains of child development at all ages. Higher scores of psychosocial stimulation was significantly associated (*p* < 0.001) with better communication development for children under 1 and 2 years of age, while greater knowledge of emergency services was significantly associated with better motor development for children under 1. Reduced exposure to environmental hazards was associated with better overall development in children of all age groups, while also being associated with better motor and communication development in children under 3. The variance explained at province level relating to the relationship between total quality of childcare and child development is high at 64.432%, 81.048%, and 85.802% for 1 year (Y), 2Y and 3Y development, respectively.

**Table 4:** Exploratory factor analysis for quality of childcare index.

Items	Psychosocial stimulation	Exposure to environmental danger	Knowledge of environmental services	Nutrition
Homemade toys	0.452			
Store bought toys	0.498			
Uses objects at home as toys	0.417			
Read books, identify objects, count and draw with child	0.751			
Read stories or sing with child	0.744			
Takes child out for a walk	0.497			
Play games with child	0.691			
Takes child to play with water in lakes		−0.234		
Lets child to play with water in lakes with other non-adults		0.232		
Lets child bath alone		0.359		
Lets child play in kitchen or bathroom		0.555		
Matches and lighters are placed within child’s reach		0.735		
Hot water or soup are placed within child’s reach		0.755		
Flammable objects such as alcohol and petrol are placed indoors		0.323		
Knows the number for police department			0.887	
Knows the number for fire departments			0.890	
Knows the number to call ambulance			0.897	
Fed child dairy products				−0.342
Fed child carbohydrates				0.690
Fed child food high in Vitamin A				0.720
Fed child eggs				0.681
Fed child meat, fish, or animal intestines				0.696
Fed child beans or nuts				0.656
Reliability correlations, Cronbach *α*	0.736	0.562	0.880	0.735

Confirmatory factor analysis model fit indices (not presented just stated in text)						
	Items	Factors	*X*^2^/2	CFI	TFI	RMSEA
4 factor model	20	4	1235.162	0.845	0.802	0.062

Multilevel analysis on factors influencing quality of childcare is further presented in Table 5. Higher education was also associated with better knowledge of emergency services, reduced exposure to environmental hazards, nutrition and better overall quality of childcare. Being left behind was also associated with receiving worse overall quality of childcare (Table 6). In contrast, higher income (>10,000 yuan) was associated with worse development in children under 1 and overall in children under 3.

**Table 5:** Association between quality of childcare and developmental score.

		Psychosocial stimulation	Environmental hazard	Knowledge of emergency services	Nutrition	Total quality of childcare score	Variance explained province level		Variance explained on household level	
6–12 M total score	Parameter	−0.004	0.355***	−0.015	0.043	0.0001	0.471***	64.432%	0.260***	35.568%
	SE	0.028	0.070	0.019	0.029	0.0001	0.077		0.060	
6–12 M motor	Parameter	−0.095***	0.011	0.058*	−0.119***	0.0001	0.098	11.543%	0.751***	88.457%
	SE	0.030	0.084	0.021	0.031	0.0001	0.137		0.144	
6–12 M communication	Parameter	0.052***	0.106***	−0.032***	0.082***	0.0001	0.0001	0.042%	0.239***	99.958%
	SE	0.016	0.041	0.011	0.016	0.0001	0.0001		0.017	
18–24 M total score	Parameter	0.017	0.070*	0.019	0.034	0.0001	0.526***	81.048%	0.123***	18.952%
	SE	0.016	0.029	0.010	0.020	0.0001	0.032		0.013	
18–24 M motor	Parameter	−0.018	0.014	0.001	−0.020	0.0001	0.033	11.340%	0.258***	88.660%
	SE	0.011	0.020	0.007	0.013	0.0001	0.021		0.023	
18–24 M communication	Parameter	0.029*	0.003	0.004	0.039***	0.0001	0.026	8.814%	0.269	91.186%
	SE	0.011	0.020	0.007	0.013	0.0001	0.021		0.023	
30–36 M total score	Parameter	−0.007	0.004	0.004	0.019	0.0001	0.556***	85.802%	0.092***	14.198%
	SE	0.015	0.021	0.009	0.017	0.0001	0.026		0.007	
30–36 M motor	Parameter	−0.010	0.034*	0.006	0.016	0.0001	0.030*	10.490%	0.256***	89.510%
	SE	0.009	0.014	0.006	0.011	0.0001	0.014		0.016	
30–36 M communication	Parameter	0.018*	0.026	0.016*	0.014	0.0001	0.027	9.153%	0.268***	90.847%
	SE	0.009	0.014	0.006	0.012	0.0001	0.014		0.016	
Total 3Y and under score	Parameter	0.003	0.036*	−0.0001	0.034*	0.0001	0.554***	77.809%	0.158***	22.191%
	SE	0.011	0.018	0.007	0.012	0.0001	0.022		0.007	
Total motor score	Parameter	−0.028***	0.030**	0.014*	−0.029***	0.0001	0.037***	9.946%	0.335***	90.054%
	SE	0.007	0.013	0.005	0.009	0.0001	0.011		0.013	
Total communication score	Parameter	0.002	0.036***	0.009*	0.024***	0.0001	0.019*	6.333%	0.281***	93.667%
	SE	0.007	0.011	0.004	0.008	0.0001	0.008		0.011	

*p < 0.05.

**p < 0.01

***p < 0.001.

**Table 6:** Association of primary care giver demographic factors and quality of childcare.

		Left behind children	Highest education of main caregiver		Age of primary caretaker	Household income			Main source of income			Variance explained province level		Variance explained on individual mother level		
			Primary	Secondary and beyond		2000–5999	6000–10,000	>10,000	Business	Casual/part time job	Other					
Psychosocial stimulation	Parameter	−0.002	0.010	−0.007	−0.0001	−0.000	0.005	−0.005	0.004	−0.001	0.002	2.818***	99.965%	0.001***		0.035%
	SE	0.027	0.008	0.009	0.0001	0.007	0.008	0.008	0.008	0.009	0.018	0.104		0.0001		
Environmental hazard	Parameter	0.037	0.054	0.101***	−0.001	−0.023	−0.071	−0.003	−0.036	0.030	0.090	0.925**	96.757%	0.031***		3.243%
	SE	0.118	0.031	0.036	0.001	0.030	0.033	0.036	0.057	0.040	0.077	0.035		0.002		
Emergency services	Parameter	−1.349***	1.608***	1.860***	−0.012***	−.243**	−0.190	−0.148	0.110	−0.183	0.211	5.172***	91.686%	0.469***		8.314%
	SE	0.423	0.104	0.117	0.004	0.111	0.122	0.128	0.207	0.144	0.277	0.206		0.026		
Nutrition	Parameter	−0.149	0.110	0.211**	0.003	0.020	−0.045	0.038	−0.032	0.082	0.180	1.679***	91.449%	0.157***		8.551%
	SE	0.244	0.060	0.067	0.002	0.064	0.071	0.074	0.120	0.083	0.160	0.067		0.009		
Total score	Parameter	−1.782**	1.277***	2.234***	−0.009	−0.241	−0.238	−0.055	0.066	−0.080	0.490	13.321***	95.361%	0.648***		4.639%
	SE	0.525	0.136	0.154	0.005	0.136	0.150	0.157	0.254	0.179	0.343	0.512		0.036		
6–12 M development	Parameter	0.091	−0.152*	−0.139*	−0.003	−0.198*	−0.223*	−0.279***	0.174	0.191	0.815***	0.082***	85.417%	0.014*		14.583%
	SE	0.313	0.054	0.058	0.003	0.080	0.090	0.091	0.233	0.150	0.233	0.012		0.005		
18–24 M development	Parameter	−0.026	0.006	−0.082*	0.0001	0.002	−0.024	−0.008	0.021	0.023	0.023	0.091***	96.809%	0.003***		3.191%
	SE	0.061	0.025	0.029	0.001	0.024	0.026	0.026	0.043	0.034	0.054	0.005		0.0001		
30–36 M development	Parameter	0.122	0.014	−0.007	0.001	−0.013	−0.005	−0.036	−0.087	−0.069*	0.007	0.063***	74.118%	0.022***		25.882%
	SE	0.150	0.022	0.024	0.001	0.027	0.030	0.031	0.050	0.032	0.066	0.004		0.002		
Total 3Y development	Parameter	0.074	−0.016	−0.029	0.0001	−0.022	−0.018	−0.052*	−0.042	−0.037	0.052	0.070***	74.468%	0.024***		25.532%
	SE	0.078	0.017	0.019	0.001	0.022	0.024	0.025	0.040	0.027	0.052	0.003		0.001		

*p < 0.05.

**p < 0.01

***p < 0.001.

## Discussion

To understand the influence of nurturing care on child development, a quality of childcare index was devised based on WHO’s Nurturing Care Framework.^7^ The four factors included in the index are (1) psychosocial stimulation, (2) exposure to environmental hazards, (3) primary caregiver knowledge of emergency services, and (4) child nutrition. Reasonable level reliability of the index and excellent model fit results suggested that the quality of childcare index can be used to assess the childcare quality in rural areas in China.

In our study, the prevalence of children at risk of developmental delays were similar (*p* = 0.661) between children left behind (89.0%) and children raised by both parents (89.6%). This differed to previous studies that identified being left behind as a significant risk factor for children under 60 M.^21^ Nonetheless, we found that children left behind received significantly worse quality of childcare, with worse psychosocial stimulation and greater exposure to environmental hazards. Furthermore, caregivers of children left behind also appeared to have worse knowledge of emergency services, acting as negative factors associated with risk of developmental delays.

Based on the 20-item scale devised, the association between quality of childcare on child development in motor, communication and problem-solving domain across different age groups were modeled using multilevel analysis. Significant intra-class correlation coefficient calculations based on the multilevel analysis for quality of childcare index suggested province-level differences in wealth, demographic factors and resources was significantly associated with quality of childcare. Although only rural regions of provinces and municipality included were sampled, significant variations in household wealth, main source of household income, highest education of primary caregiver and household income were still identified across the 12 provinces and municipalities included. These variations were further reflected in multi-level analysis, where variance explained on a province level remained high across all domains and age ranges of development assessed.

Similar variations between provinces were also found in previous studies that identified increased inequality in income between provinces and regions in the last 60 years.^22^ This may be explained by the difference in the pace of urbanization and economic growth in different rural regions and provinces, as the extent of urbanization and economic growth are dependent on regional development policies.^22,23^ Provinces that have been urbanized with greater economic growth will have better education and job opportunities that are further associated with better household income and quality of childcare. In the current study, we found that a caregivers in high GDP provinces had better education and higher household income as opposed to caregivers of children residing in the lowest GDP provinces. Thus, difference in education and job opportunities between provinces may be a significant factor influencing child development and quality of childcare.

In this study, we found a greater proportion of children left behind from higher-GDP provinces as compared to lower-GDP provinces. This was further reflected in the difference in primary caregiver as more children in higher-GDP provinces had their grandparents act as their primary caregivers as compared to children who were cared by parents from lower-GDP provinces. While parental migration to urban regions for work may improve family socioeconomic circumstances, the benefits of economic gain in the absence of adequate parental care has been questioned.^8,10,12,24^ Although a greater percentage of households are classified within the highest bracket of household income (>10,000 yuan), more children in higher-GDP provinces were left behind with their grandparents acting as the primary caregiver. A trade-off exists between increased income and reduced parental involvement with heightened emotional stress from being separated with their parents.^8,24^ Similarly, we found a higher proportion of children left behind at high risk of developmental problems in higher-GDP provinces as compared to lower-GDP provinces. Similar proportions of children at high developmental risks were identified between children left behind and children raised by both parents in middle and lower-GDP provinces. However, the proportion of children raised by both parents at high developmental risks was significantly lower in higher-GDP provinces. These results suggested that while moving to urban regions for work may improve the SES for the household, benefits of increased income may be limited in child development in the absence of nurturing care.

To achieve healthy development, primary caregivers must be able to provide nurturing care encompassing adequate nutrition, opportunities for early learning, good health, responsive caregiving, and a secure and safe environment based on WHO’s framework.^7^ However, nurturing care is difficult to achieve for children left behind. Lack of communication and contact from their parents often lead to feelings of loneliness and disruptions to parent-child attachment, resulting in poor socio-emotional development.^8,24^ Importance of psychosocial stimulation in nurturing care is further highlighted as higher psychosocial stimulation score predicted better communication development in this study. Although multi-level analysis found psychosocial stimulation to be negatively associated with motor development, the results were disregarded as questions included in the psychosocial stimulation subscale were related to reading, counting, singing and other activities irrelevant to motor development. When demographic differences across provinces and municipalities were accounted for in analysis, we found that being left behind was significantly associated with worse psychosocial stimulation, suggesting reduced early learning opportunities and interactions with primary caregivers. This may be explained by the low education status of the primary caregivers, as more than 20% of the primary caregivers of children left behind received no education, while around half of them only had primary education. When demographic differences between provinces were accounted for, higher education was still significantly associated with better quality of childcare in all aspects. In contrast, higher income did not significantly predict better overall quality of childcare. Although statistically significant, the difference in household wealth between children left behind and children raised by both parents were small, suggesting that education status of primary caregivers is a more important factor that contribute to quality of childcare.

In this study, primary caregivers with higher education were significantly associated with reduced child exposure to environmental hazards and significantly better knowledge of emergency services. This suggests that primary caregivers with limited education may have poor knowledge in environmental hazards that puts children at risk of unintentional injury.^25^ Hazards in the home environment has a direct impact on unintentional injuries as young children lack risk perception, making quality adult supervision and safe household environment critical to prevent unintentional injuries.^25^ Storage of flammable objects and supervision around chemical and fire hazards are critical to prevent serious injuries including burns and poisoning.

Following the injury, primary caregivers with low education do not have the required knowledge to reach the correct emergency services including police, fire department and ambulance services, which puts the child at further risk for poor outcomes. This is in line with the results of the study which found reduced exposure for environmental hazards and better knowledge of emergency services as significant predictors for better motor development. A safe household without hazards allow children to explore and engage in early learning opportunities by playing with safe and age-appropriate toys, while better knowledge of emergency services allows for timely treatment during injuries, contributing to nurturing care to ensure health development.^7^ Given the importance of caregiver education on quality of childcare across multiple domains, China has issued guidelines in 2016 to develop caregiver education programs to improve caregiver knowledge on feeding, daily care, child safety and development in multiple provinces.^26^ The effectiveness of parenting or caregiver programs involving parent–child classes, lectures and online platforms have showed success in improving childcare knowledge.^26^

Nutrition is another important component of nurturing care that was significantly associated with better child development, especially in communication in this study. Optimal nutrition is critical to support brain metabolism, especially in the first three years of life that is deemed as a sensitive period for brain development.^11^ In the absence of adequate nutrition, children are at risk of developmental delays and disabilities due to the lack of neurobehavioral development.^11^ Limited brain development in the first few years of life may limit the child’s ultimate brain capacity and have long-term consequences in poor education and job potential.^11,27^

## Conclusion

Differences in skillset, job opportunities, and income influence whether a parent migrate to urban regions for work, leaving their children behind. Migration of children along with working parents are difficult not only due to financial barriers in moving into urban regions, household registration systems in China also prevent children from doing so.

While parental migration from rural to urban regions for work may provide better income for the household, significant detriments to quality of childcare and development exists. Children who were left behind had the lowest mean score in quality of childcare, while being left behind was associated with worse overall child development. Education status of the primary caregiver appeared to be the most important factor in predicting quality of childcare and development. Furthermore, despite the sampled population being only from rural regions, significant differences existed across rural communities in different provinces. As one of the most marginalized and at-risk population from poor quality of childcare and poverty, changes in policies are required to reduce wealth and geographical inequalities that drive parents to leave their child and migrate to urban regions for work. For children left behind, interventions such as government supported parenting programs for their primary caregivers may help improve awareness in environmental hazard, knowledge in emergency services, child nutrition, and importance of early psychosocial stimulation to achieve nurturing care recommended by WHO.

## Data Availability

The datasets generated during and/or analyzed during the current study are available from the corresponding author on reasonable request.
